# Supplementary material for: DMRforPairs: identifying Differentially Methylated Regions between unique samples using array based methylation profiles
Source: BMC Bioinformatics. 2014 May 15;15:141. doi: 10.1186/1471-2105-15-141 (PMC4046028; doi:10.1186/1471-2105-15-141)
Supplement: Additional file 2 — DMRforPairs output for the comparison of A431-MCF7 and NA17018-NA17105. Please start from the HTML files in each folder. Available via the BMC Bioinformatics website. [file 1471-2105-15-141-S2.zip › 1394847754114233_MOESM2_ESM/A431_MCF7/figures/975.pdf]

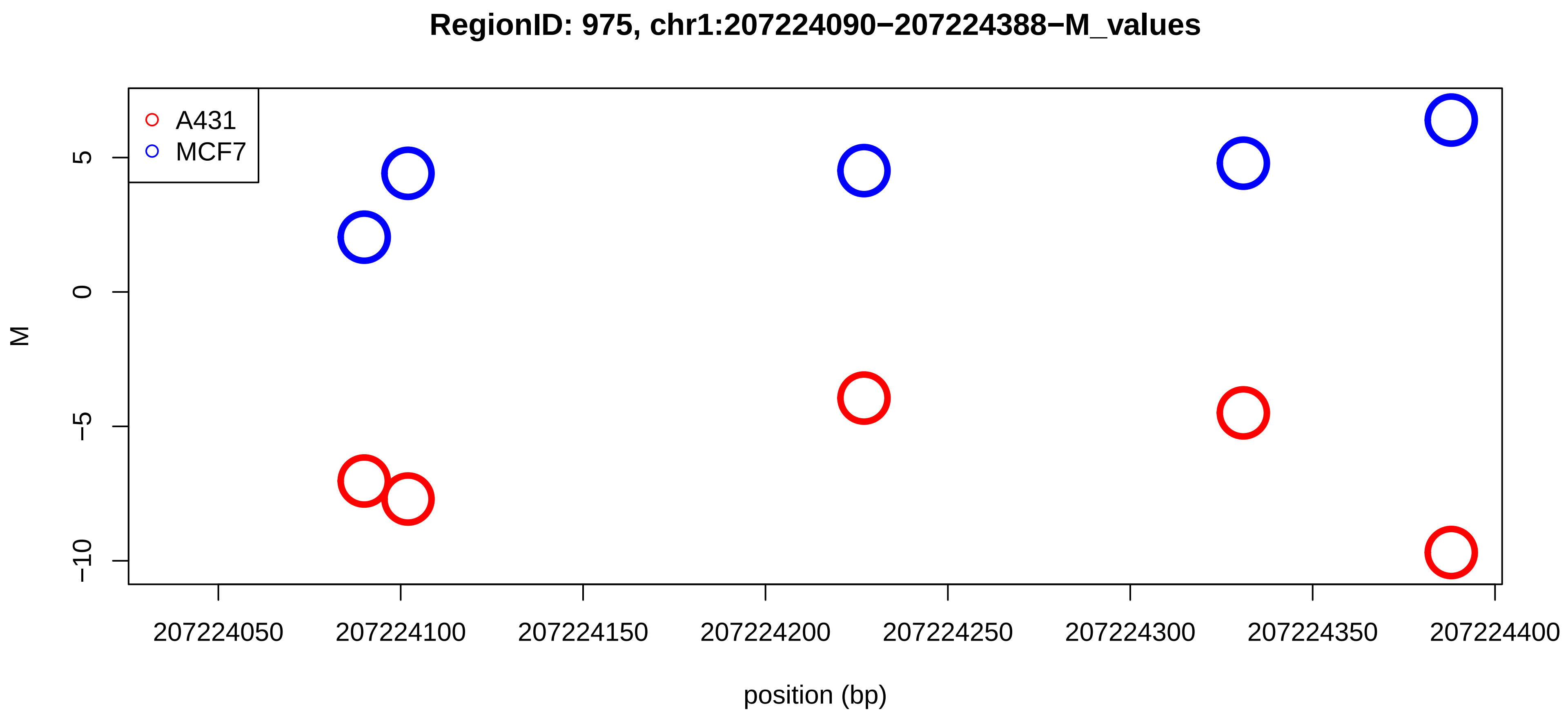
RegionID: 975, chr1:207224090−207224388−M_values
A431
MCF7
M
5
0
−5
−10
207224050
207224100
207224150
207224200
207224250
207224300
207224350
207224400
position (bp)

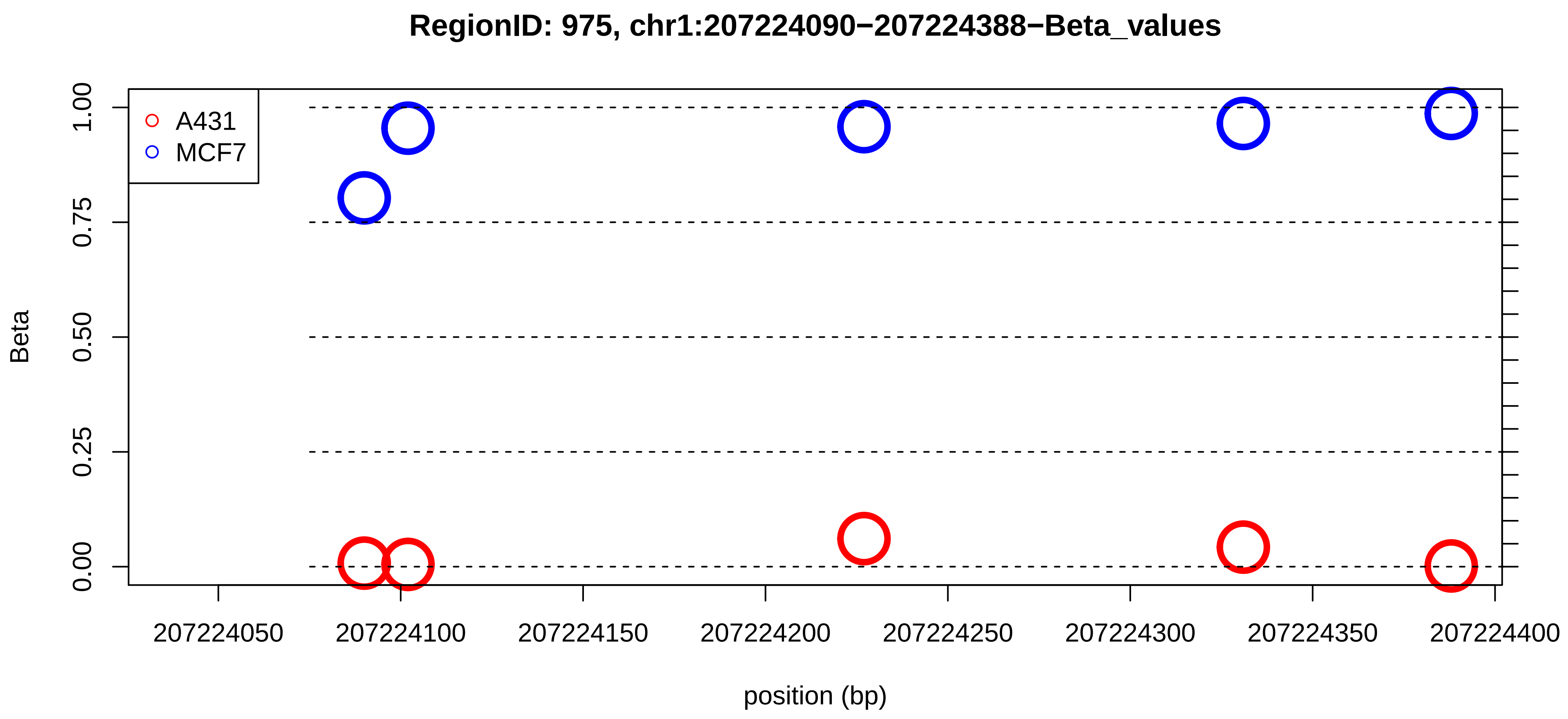
RegionID: 975, chr1:207224090−207224388−Beta_values
A431
MCF7
Beta
1.00
0.75
0.50
0.25
0.00
207224050
207224100
207224150
207224200
207224250
207224300
207224350
207224400
position (bp)

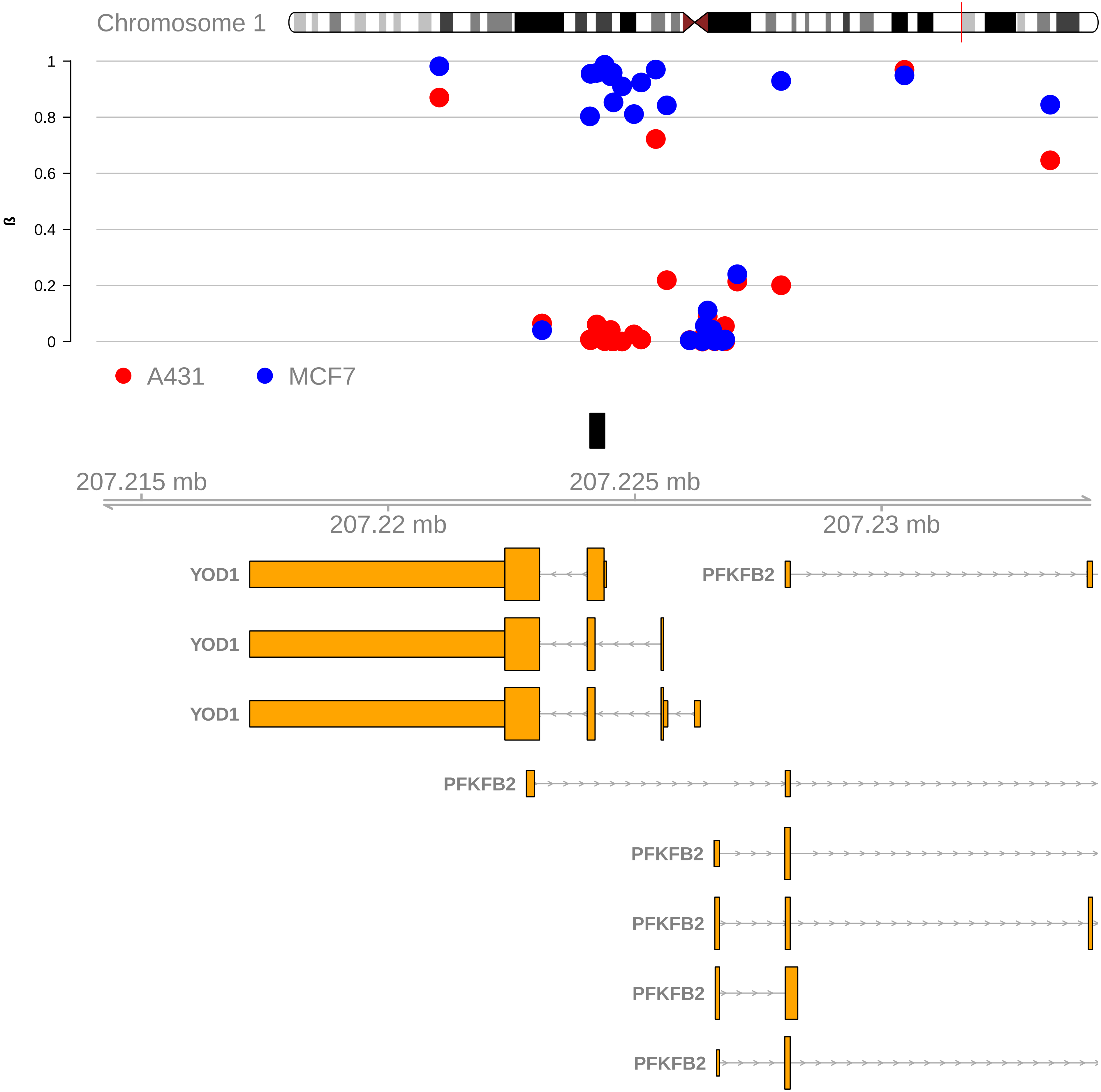
Chromosome 1
ß
1
0.8
0.6
0.4
0.2
0
A431
MCF7
207.215 mb
207.225 mb
207.22 mb
207.23 mb
YOD1
PFKFB2
YOD1
YOD1
PFKFB2
PFKFB2
PFKFB2
PFKFB2
PFKFB2
